# Supplementary material for: SyNDI: synchronous network data integration framework
Source: BMC Bioinformatics. 2018 Nov 6;19:403. doi: 10.1186/s12859-018-2426-5 (PMC6219086; doi:10.1186/s12859-018-2426-5)
Supplement: Supplementary file 5 — Files of the example presented in Additional file 4. This additional file is a zip package that contains all files used in Additional file 4. Detailed descriptions of each of these files are presented in a README file included in this zip package. (GZ 30411 kb) [file 12859_2018_2426_MOESM5_ESM.gz › SyNDITools/Meme2Fimo/meme_4.9.0_4.tar.gz!/meme_4.9.0/doc/getsize.xml]

getsize


# getsize

**Usage:** getsize <datafile> [options]

**Description:**

`getsize` prints statistics about the sequences in
`<datafile>`.

**Input:**

- A file in FASTA format.
  If the filename is given as "-", then the program reads from
  standard input.

**Output:**

- Writes statistics about the sequences to `stdout`.

**Options:**


**Bugs:** None known.

**Author:** Tim Bailey
